# RANBP10 promotes glioblastoma progression by regulating the FBXW7/c-Myc pathway

**DOI:** 10.1038/s41419-021-04207-4

**Published:** 2021-10-20

**Authors:** Jianbing Hou, Yudong Liu, Pan Huang, Yutao Wang, Dakun Pei, Ruoyue Tan, Yundong Zhang, Hongjuan Cui

**Affiliations:** 1 State Key Laboratory of Silkworm Genome Biology, Southwest University, 400716 Chongqing, China; 2 Cancer Center, Reproductive Medicine Center, Medical Research Institute, Southwest University, 400716 Chongqing, China; 3 Department of Neurology, the Third Affiliated Hospital of Chongqing Medical University, Chongqing, China

**Keywords:** Targeted therapies, CNS cancer, Prognostic markers

## Abstract

RAN binding protein 10 (RANBP10), a ubiquitously expressed and evolutionarily conserved protein, as a RAN-GTP exchange factor (GEF) to regulate several factors involved in cellular progression. Previous studies showed that RANBP10 was overexpressed in prostate cancer cells and was responsible for androgen receptor (AR) activation. However, the biological function of RANBP10 in glioblastoma (GBM) has not been studied. Here, we found that RANBP10 was overexpressed in GBM, and high RANBP10 expression was closely linked to poor survival of patients with GBM. Downregulation of RANBP10 significantly inhibited cell proliferation, migration, invasion, and tumor growth of GBM cells. In addition, we revealed that RANBP10 could suppress the promoter activity of FBXW7, and thereby increase the protein stability of c-Myc in GBM cells. Silencing of FBXW7 in RANBP10-knockdown GBM cells could partly negate the effects induced by RANBP10 downregulation. Taken together, our findings established that RANBP10 significantly promoted GBM progression by control of the FBXW7–c-Myc axis, and suggest that RANBP10 may be a potential target in GBM.

## Background

Diffuse gliomas, a heterogeneous group of central nervous tumors, are characterized by rapid proliferation, intensive infiltration, and resistance to treatment. Glioblastoma multiforme (GBM), grade IV gliomas classified by the World Health Organization (WHO), is the most frequent, aggressive, and lethal brain malignancy in adults [1, 2]. Despite enormous efforts for clinical treatment for GBM, the prognosis of tumor patients only improved 2.5 months after diagnosis [3, 4]. The current challenge is considered key to investigate the mechanism of driving GBM progression and treatment resistance of GBM [5, 6].

RANBP10, a ubiquitously expressed and evolutionarily conserved protein, is initially identified as RAN binding proteins and functions as a RAN-GTP exchange factor (GEF) [7, 8]. RANBP10 exerts important functions by interacting with several proteins, such as MET, RAN, YPEL5 (Yippee like 5), Protein Kinase C (PKC), and β1-tubulin [8–11]. In addition, RANBP10 also acts as a transcriptional regulator for the viral genes and androgen receptor (AR) [12, 13]. Recent studies have demonstrated that RANBP10 played important roles in the cell cycle, spindle assembly, the reproductive system, and tumor progression [8, 10, 13, 14]. In AR-positive prostate cancer cells, RANBP10 was highly expressed and was responsible for the activation of AR, thereby contributed to prostate cancer development and progression [13]. Furthermore, RANBP10 might function in the DNA Damage Response (DDR) of cancer cells due to the post-translational modification following genotoxic stress [7]. However, the biological function of RANBP10 in tumors, especially in GBM has not been studied.

The c-Myc oncogene (gene locus: 8q24.12-q24.13) acts as a strong and pluripotent regulator during normal cell development and progression [15]. Deregulation and/or mutation of c-Myc are present in most human neoplasms and consider to be correlated with poor prognosis of tumor patients [16]. Ectopic c-Myc expression in human malignancy tumors induces the excessive activation of its downstream genes and/or signaling pathways, which contributes to cell growth and proliferation, metastasis, cell metabolism, and tumorigenesis [16, 17]. c-Myc expression is tightly controlled by a series of mechanisms including acetylation, phosphorylation, transcriptional regulation, and ubiquitin-dependent proteolysis [16, 18].

F-box and WD repeat domain containing 7 (FBXW7), also named as FBW7, hCDC4, and AGO, is a crucial E3 ubiquitin ligase for targeting several important factors and proto-oncogenes, including c-Jun, CyclinE, MCL-1, Notch, and c-Myc [19]. Increasing evidence have demonstrated that mutational and allelic loss of FBXW7 in human cancers were implicated in processes of tumor development and progression [20–23]. Reducing of FBXW7-mediated c-Myc proteasome degradation was reported to give rise to c-Myc protein-level upregulation and to associate with a poor prognosis in tumor patients [22]. Therefore, tight regulation of FBXW7 expression is required.

In this study, we found that RANBP10 promoted GBM cell proliferation, migration, invasion, and tumor growth. Our study indicated that that RANBP10 elevated the stability of c-Myc protein by suppression of FBXW7-transcriptional activity. Collectively, our data demonstrated that RANBP10 may be a potential therapeutic target.

## Materials and methods

### Cell culture and reagents

GBM cell lines (U-118 MG, A172, U-87 MG, U-251 MG, and LN-229) and normal astroglia cells (SVGP12) were obtained from the American Type Culture Collection (ATCC) and cultured in DMEM medium supplemented with 10% fetal bovine serum (FBS) and 1% penicillin and streptomycin (P/S). The 293FT cell line was culture as previously described [2]. All cell lines were tested mycoplasma-negative.

The FBS, antibiotics, and DMEM media were obtained from Thermo Fisher Scientific (MA, USA). Crystal violet and Dimethyl sulfoxide (DMSO) were purchased from Sigma (MO, USA). The RANBP10 (21107), E-cadherin (20874), p21 (10355), CyclinD1 (60186), Myc (60003), and Tubulin (11224) antibodies were obtained from Proteinch (Wuhan, China). The c-Myc (32072) and FBXW7 (109617) were obtained from Abcam (Shanghai, China); The CDK4 (12790), CDK6 (13331), N-cadherin (13116), Flag (14793), and HA (C29F4) antibodies were obtained from Cell Signaling Technology (MA, USA).

### Cell transfection

Sequences of the shRANBP10 and shFBXW7 were listed as below:

Scramble: AGCACACTAGAACCATGTGAA

shRANBP10#1: CAAGTTGGTGATAGCTTATTA

shRANBP10#2: AGATTGTGGACGCCAACTTTG

shRANBP10#3: CAAAGGAAGAGATGGTTACAT

shFBXW7: CCAGAGAAATTGCTTGCTTTA

The full length of RANBP10 and FBXW7 was obtained from Youbio (Wuhan, China). Cell transfection and lentivirus were produced as previously described [24].

### Immunohistochemistry staining

Tumor slides were incubated for 3 h at 60 °C and then deparaffinized. After rehydration and antigen retrieval, the tumor sections were blocked with 3% H_2_O_2_ for 15 min at room temperature and goat serum for 2 h at 37 °C. The sections were sequentially incubated with primary antibodies and secondary antibodies. The immunohistochemistry results were analyzed as previously described [24].

### MTT assays

For the MTT assays, cells (1000 cells/well) were seeded into the 96-well plate, and cell proliferation was detected using MTT according to the manufacturer’s instructions. The absorbance was measured using a microplate reader (CA, USA).

### BrdU staining

Cells (2 × 10^4^) were seeded into a 24-well plate. After incubation with BrdU (10 μg/ml, Sigma) for 1 h and paraformaldehyde (PFA, 4%) for 20 min, cells were treated with HCL (1 mol/l) for 15 min and then blocked with goat serum for 2 h. The cells were incubated with primary antibodies overnight at 4 °C and secondary antibodies for 2 h at room temperature, followed by incubation with DAPI for 30 min. BrdU-positive cells were examined using the fluorescence microscope (Nikon 80i; Tokyo, Japan).

### Flow cytometry

For the cell cycle analysis, cells were harvested and then incubated with 75% ethanol for 24 h at 4 °C. The cell lysate was washed by PBS buffer and stained with Propidium Iodide (BD, San Jose, CA, USA) and RNaseA (Sigma) at room temperature for 1 h, and then analyzed with the FACS C6 flow cytometry (BD, USA).

For cell apoptosis analysis, cells were harvested and washed with cold PBS buffer twice. The cell was incubated with binding buffer containing PI and Annexin-V-APC (BD, USA) at room temperature, and then analyzed with the FACS C6 flow cytometry.

### Transwell assay

The migration/invasion assay was performed using the Boyden chambers (8-μm pore size, Corning). In total, 2 × 10^5^ cells were seeded in transwell (Corning, USA) with 100 µl serum-free medium, and 500 µl complete medium was added into the lower chamber. After 9 h, cells in the upper chamber were erased and cells in the lower chamber were fixed by 4% paraformaldehyde followed by 0.1% crystal violet staining. The procedure for the invasion assay was similar to that for the migration assay, except that the transwell membranes were pre-coated with Matrigel (R&D Systems, USA) and the cells were incubated for 18 h at 37 °C in a 5% CO_2_ atmosphere. The stained cells were examined in the microscope and counted by ImageJ.

### Western blot and Co-IP

For the western blot assay, cells were harvested and lysed in RIPA lysis buffer (Beytome, Shanghai, China) supplemented with the protease inhibitor cocktail. Proteins were resolved by SDS-PAGE and then transferred to PVDF membranes. After being blocked with bovine serum albumin (BSA, 5%) for 2 h at room temperature, the membranes were sequentially incubated with primary antibodies overnight at 4 °C and secondary antibodies for 2 h at room temperature. Proteins were visualized by the Image Reader (Clinx Science Instruments Co., Ltd., Shanghai, China).

For the Co-IP assay, cells were harvested and lysed in the western blot and IP lysis buffer (Beytome, Shanghai, China) and then performed as previously described [2].

### Quantitative and RT-PCR

The total RNA was extracted from the cultured cells using the TRIzol reagent (Thermo Fisher Scientific) according to the manufacturer’s instructions. Complementary DNA (cDNA) was harvested from total RNA using the GoScript cDNA Synthesis Kit (BioRad, #170-8891). RT-PCR was then performed using the Roche LightCycler Real-Time PCR System and specific primers (Table 1).

**Table 1:** Primer pairs for real-time PCR.

RANBP10-F	GGCTCAAGGCGTCAACATGA
RANBP10-R	GGAGCAGAACGAATGCCCAT
c-Myc-F	GTCAAGAGGCGAACACACAAC
c-Myc-R	TTGGACGGACAGGATGTATGC
CyclinD1-F	GCTGCGAAGTGGAAACCATC
CyclinD1-R	CCTCCTTCTGCACACATTTGAA
CDK4-F	ATGGCTACCTCTCGATATGAGC
CDK4-R	CATTGGGGACTCTCACACTCT
CDK6-F	CCAGATGGCTCTAACCTCAGT
CDK6-R	AACTTCCACGAAAAAGAGGCTT
E-cadherin-F	ATTTTTCCCTCGACACCCGAT
E-cadherin-R	TCCCAGGCGTAGACCAAGA
N-cadherin-F	AGCCAACCTTAACTGAGGAGT
N-cadherin-R	GGCAAGTTGATTGGAGGGATG
p21-F	TAATTGGGGCTCCGGCTAACT
p21-R	TGCAGGTCGCTTCCTTATTCC
FBXW7-F	TAGAACCCCAGTTTCAACGAGA
FBXW7-R	GCCAACTCTTTAGGGAGCAAT
GAPDH-F	GGAGCGAGATCCCTCCAAAAT
GAPDH-R	GGCTGTTGTCATACTTCTCATGG

### Chromatin immunoprecipitation

Chromatin was isolated from 2 × 10^7^ U-87 MG/Vector and U-87 MG/Flag-RANBP10. ChIP assays were performed using the EZ-ChIP^TM^ kit (Millipore, CA, USA), and then detected according to the manufacture’s protocol. The primers used in ChIP assays are listed in Table 2.

**Table 2:** Primer pairs for ChIP assays.

FBXW7-1450/-1007-F	GCTTTGATTTGCATTTCCCTAAT
FBXW7-1450/-1007-R	TGAACTATCCATTCACAGTGCTCA
FBXW7-1036/-729-F	GTTGATTGAGCACTGTGAATGGA
FBXW7-1036/-729-R	TGCAAATCCCTAGTGCCTAATTT
FBXW7-753/-446-F	AGAAATTAGGCACTAGGGATTTGC
FBXW7-753/-446-R	CTCTCTTTCCCTTTTCCATACACAC
FBXW7-450/-116-F	GAGAGCACACACTTGAAAATAGTGC
FBXW7-450/-116-R	ATGTGAACACAACCAAAGCAGG
FBXW7-137/173-F	CCTGCTTTGGTTGTGTTCACAT
FBXW7-137/173-R	AAAACTCCTCTTGGTTGACGAATAC

### Turnover assay and ubiquitination assay

For the turnover assay, indicated cells were incubated with a final concentration (100 μg/ml) of CHX and then harvested at the indicated time points. Proteins were extracted from the cells and analyzed by western blotting.

For the ubiquitination assay, indicated cells were incubated with or without MG132 (50 μg/ml, Selleck, Houston, TX, USA) for 8 h, and then harvested and analyzed by Co-IP.

### Luciferase reporter assay

Cells were transfected with shRANBP10 or RANBP10 together with the indicated reporter plasmids. The promoter activity was detected using the Dual-Luciferase^®^ Reporter Assay System (Promega, #E1910) according to the manufacturer’s instructions. The promoter fragments of FBXW7 were obtained from Wuhan GeneCreate Biological Engineering Co., Ltd.

### Colony-formation assay

For the colony-formation assay, cells were trypsinized, counted, and plated on a six-well plate. After two weeks, the colonies were incubated with 4% PFA for 20 min and crystal violet for 15 min and then counted.

### Xenograft assay

The procedures were performed as described previously [2]. Briefly, NOD/SCID mice (4-week-old female) were purchased from Beijing Animal Research Center and were housed in the SPF room. In total, 1 × 10^5^ GBM cells (U-87 MG) stably transfected with Scramble or shRANBP10 were intracranially injected slowly into the brain of each mouse. We divided the mice into two groups, one group (six mice for each subgroup) was used for H&E staining, the other group (six mice for each subgroup) was monitored for survival. Isoflurane anesthesia (MAC 1.6%, Reyward Life Technology, Shenzhen, China), an inhalation general anesthesia, was used to induce mice to enter an anesthetized state faster and recover quickly. The anesthesia induction is safe, rapid, stable, fast recovery, and no nervous system excitatory effects. The mice were sterilized with medical alcohol (75%) after injection and were weighed every 2 days. Before the tumors were collected, isoflurane anesthesia was used to reduce the pain of the mice. Then, the mice were killed by cervical dislocation and the brains were harvested. The tumor volumes were measured using length (a) and width (b) and calculated using the equation: V = ab^2^/2. The data represent the means ± SD. of six mice. Randomization and single blinding were used for measurement. The bodies were stored at −20 °C and then incinerated by Laibite Biotech Inc. (Chongqing, China). All animal studies were approved by the Institutional Animal Care and Use Committee of Southwest University.

### Patient data analysis and patient tumor tissues

Bioinformatics analyses were performed using the CGGA database (http://www.cgga.org.cn/), Oncomine database (https://www.oncomine.org/), and the Human Protein Atlas (https://www.proteinatlas.org/). Clinical samples were obtained from Chaoying Biotechnology Co., Ltd. (Henan, China). Tissue analysis was approved by the Ethics Committee of the Southwest University of China. All the patients provided written informed consent to participate.

### Gene set enrichment analysis (GESA)

To determine whether RANBP10 expression was correlated with the biological processes and/or signaling pathway in GBM, GSEA (version 4.0.3) was used. The CGGA database was downloaded from the Chinese Glioma Genome Atlas (http://www.cgga.org.cn/). The gene sets were obtained from the Molecular Signatures Database (MsigDB, http://software.broadinstitute.org/gsea/index.jsp).

### Statistical analysis

All experiments were carried out in triplicates and statistical parameters including the sample size and the significance analysis are specified in figure legends. The data were expressed as mean ± SD. Two-tailed Student’s *t* test was performed to analyzed significance in an interval of 95% confidence level, and a value of *P* < 0.05 was considered statistically significant, *P < 0.05, **P < 0.01, ***P < 0.001.

## Results

### RANBP10 was highly expressed in GBM and was associated with poor prognosis of GBM patients

**Fig. 1:**
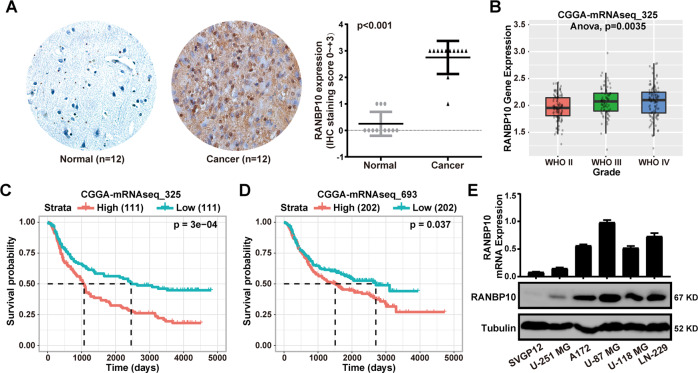
RANBP10 was highly expressed in GBM and was associated with the poor prognosis of GBM patients. **A** Immunohistochemical analysis of RANBP10 expression in 12 paired samples of glioblastoma and normal brain tissue, *P* < 0.001. **B** Box plot of RANBP10 expression levels in WHO II, WHO III, and WHO IV glioma set with the log-rank test *P* values indicated. **C**, **D** Kaplan–Meier analysis of overall survival using data from the CGGA-mRNAseq-325 and CGGA-mRNAseq-693 dataset and *P* values were indicated. **E** RT-PCR analysis and western blot analysis of RANBP10 expression in normal SVGP12 cells and glioblastoma cell lines. The data were expressed as mean ± SD. Student’s *t* test was performed to analyzed significance.

Overexpression of RANBP10 was found in several cancer types, including brain and CNS cancer, gastric cancer, kidney cancer, and prostate cancer (Supplementary Fig. S1A). In addition, the Human Protein Atlas database analysis showed that RANBP10 was commonly expressed in malignant tumors (Supplementary Fig. S1B). To determine whether the expression of RANBP10 was a prognostic factor for GBM, we performed the immunohistochemistry assays in 12 paired samples and the results indicated that RANBP10 expression was higher in GBM than the adjacent normal brain tissues (Fig. 1A). Indeed, CGGA and Oncomine database analysis showed that RANBP10 was clearly increased in malignant glioma (Fig. 1B and Supplementary Fig. S2). In addition, we analyzed the survival data from the CGGA database and found that RANBP10 high expression was implicated in the poor prognosis of glioma patients (Fig. 1C, D). To further investigate the role of RANBP10 in glioma, we analyzed the characteristics of glioma patients related to RANBP10 expression based on the CGGA database and the data demonstrated that RANBP10 expression was dramatically associated with grade and 1p19q_Codeletion_status in glioma (Table 3). Then, RT-PCR and western blot analysis were performed and indicated that RANBP10 expression was higher in GBM cells lines than the normal astroglia cells (SVGP12) (Fig. 1E). Collectively, these results demonstrated that RANBP10 was highly expressed in GBM and high RANBP10 expression was associated with a poor prognosis of GBM patients.

**Table 3:** Correlation of RANBP10 expression with clinicopathological variables in CGGA datasets.

Clinicopathological features		Cases	RANBP10 expression		*F*	*P*
			Low	High		
Age	≦42	167	88	79	0.136	0.712
	>42	158	75	83		
Gender	Male	203	103	100	0.699	0.404
	Female	122	60	62		
Grade	WHO II	103	69	34	5.773	0.003
	WHO III	79	37	42		
	WHO IV	139	57	82		
IDH mutation status	Mutation	175	89	86	0.139	0.709
	Wildtype	149	73	76		
1p19q_Codeletion_status	Non-codel	250	115	135	9.425	0.002
	Codel	67	44	23		
PRS type	Primary	229	123	106	0.123	0.884
	Secondary	30	12	18		
	Recurrent	62	28	34		

### Silencing of RANBP10 inhibited cell proliferation, migration, and invasion of GBM cells

**Fig. 2:**
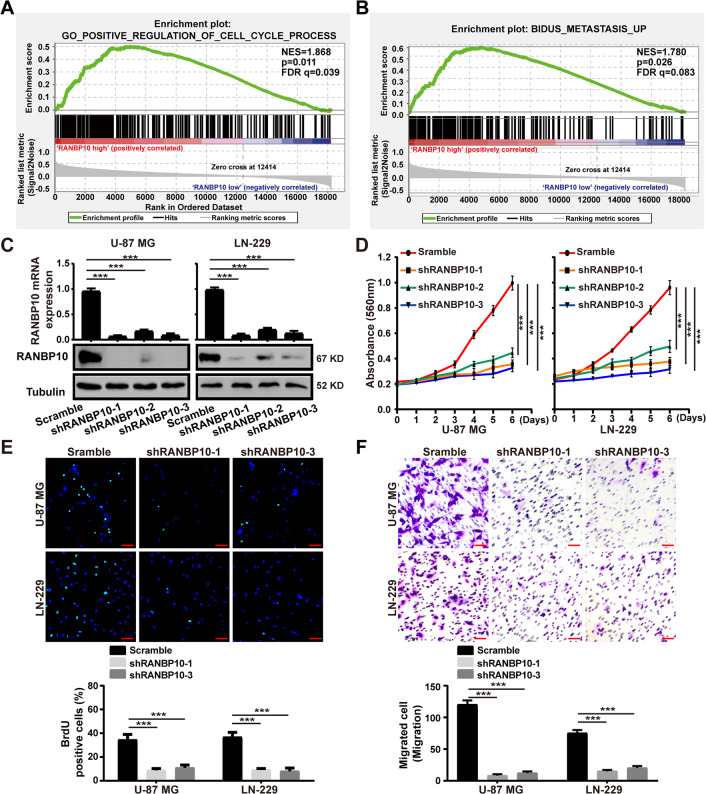
Silencing of RANBP10 inhibited cell proliferation, migration, and invasion of GBM cells. **A**, **B** GSEA enrichment analysis of cell cycle process and metastasis signatures in RANBP10 high expression versus RANBP10 low expression CGGA gliomas, Normalized enrichment score (NES), *P* values, and false discovery rate (FDR) were indicated. **C** RT-PCR analysis and western blot analysis were used to detect the mRNA and protein expression level of RANBP10 in RANBP10-knockdown and control cells. **D** Cell proliferation of RANBP10-knockdown and control cells were examined by MTT assay. **E** BrdU staining assays were performed to detect the amount of DNA synthesis in RANBP10 downregulation and control cells. Scale bar = 50 μm. **F** Cell migration of RANBP10-knockdown and control cells were examined by transwell assay. Scale bar = 50 μm. The data were expressed as mean ± SD. Student’s *t* test was performed to analyzed significance. *P < 0.05, **P < 0.01, ***P < 0.001.

To determine the importance of RANBP10 in GBM progression, we performed the GESA analysis using the CGGA database and found that RANBP10 high expression was positively associated with cell cycle and metastasis in glioma (Fig. 2A, B). Then, we knocked down RANBP10 using three shRNA sequences, shRANBP10 #1, #2, and #3. RT-PCR and western blot analysis indicated that the #1 and #3 shRNA sequence most successfully knocked down RANBP10 expression at the mRNA and protein level (Fig. 2C). Then, we examine the effect of RANBP10 downregulation on cell proliferation, and the MTT and BrdU assays indicated that RANBP10 silencing dramatically inhibited cell growth of U-87 MG and LN-229 cells (Fig. 2D, E). We then explored whether RANBP10 knockdown could induce cell death, we stained U-87 MG and LN-229 cells with PI and annexin-V and then analyzed them with flow cytometry. The results demonstrated that the silencing of RANBP10 had no significant influence on the apoptosis in glioblastoma cells (Supplementary Fig. S3). Then, flow cytometry analysis was performed and indicated that downregulation of RANBP10 could induce cell cycle arrest at the G1 phase (Supplementary Fig. S4). To explore the effect of RANBP10 downregulation on cell migration and invasion, transwell assays were performed and the results showed that RANBP10 knockdown significantly suppressed cell migration and invasion of U-87 MG and LN-229 cells (Fig. 2F and Supplementary Fig. S5). Taken together, these findings indicated that RANBP10 downregulation inhibited cell proliferation, migration, invasion of GBM cells.

### RANBP10 recovery restored the cell proliferation, migration, and invasion of RANBP10-knockdown GBM cells

**Fig. 3:**
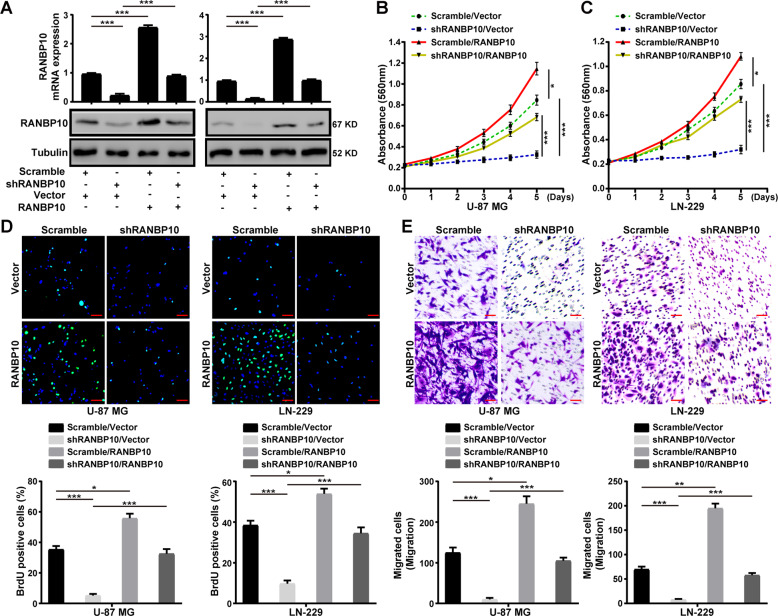
RANBP10 recovery restored the cell proliferation, migration, and invasion of RANBP10-knockdown GBM cells. **A** Western blot and RT-PCR analysis were performed to detect the expression of RANBP10 in the indicated cells. **B**, **C** MTT assays were performed to examine the effect of RANBP10 overexpression on the proliferation of RANBP10-knockdown and control cells. **D** BrdU assays were performed to examine the effect of RANBP10 overexpression on the DNA synthesis ability of RANBP10-knockdown and control cells. Scale bar = 50 μm. **E** Transwell assays were used to detect the effect of RANBP10 overexpression on the migration of RANBP10-knockdown and control cells. Scale bar = 50 μm. The data were expressed as mean ± SD. Student’s *t* test was performed to analyzed significance. *P < 0.05, **P < 0.01, ***P < 0.001.

To further confirm the functional role of RANBP10 in cell proliferation, migration, and invasion of GBM cells, we overexpressed RANBP10 in control (Scramble) and RANBP10-knockdown (shRANBP10#1) U-87 MG and LN-229 cells (Fig. 3A). MTT and BrdU assays indicated that RANBP10 overexpression could restore cell proliferation in the RANBP10-knockdown U-87 MG and LN-229 cells (Fig. 3B–D). Next, transwell assays demonstrated that RANBP10 overexpression could rescue cell migration and invasion of RANBP10-knockdown U-87 MG and LN-229 cells (Fig. 3E and Supplementary Fig. S6). Taken together, these data demonstrated that RANBP10 was essential for cell proliferation, migration, invasion of GBM cells.

### RANBP10 stabilized c-Myc protein by mediating its ubiquitination degradation

**Fig. 4:**
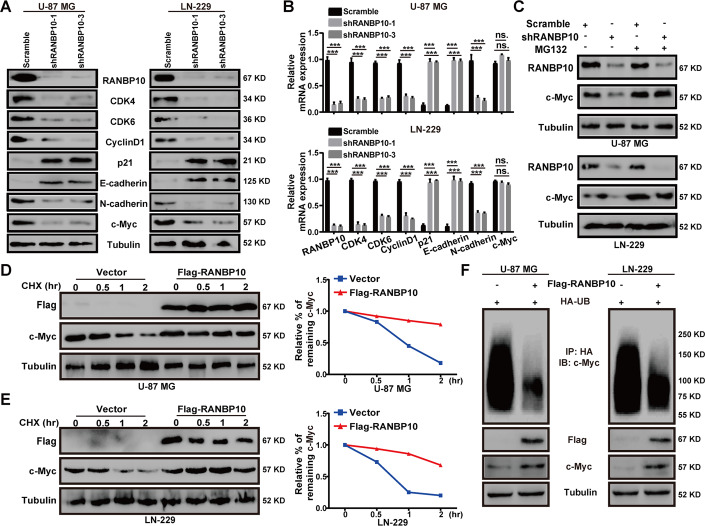
RANBP10 stabilized c-Myc protein by mediating its ubiquitination degradation. **A**, **B** Western blot analysis and RT-PCR analysis were performed to detect the expression of some G1 cell regulatory proteins and metastasis-related proteins in RANBP10-knockdown and control cells. **C** Proteins were harvested from the RANBP10-knockdown and control cells that had been treated with or without MG132 for 8 h. Western blot analysis was used to examine the protein level of c-Myc. **D**, **E** RANBP10 overexpression and control cells were treated with CHX (100 μg/ml) for the indicated times, and then were harvested and detect the c-Myc turnover rate through western blot analysis. **F** The indicated plasmids were transfected into GBM cells, and MG132 was added to the cells before harvested. The ubiquitinated c-Myc proteins were pulled down with anti-HA antibody and immunoblotted with ant-c-Myc antibody. The data were expressed as mean ± SD. Student’s *t* test was performed to analyzed significance. *P < 0.05, **P < 0.01, ***P < 0.001.

To explore the molecular mechanisms that RANBP10 regulated GBM progression, we detected the protein and mRNA expression of some G1 cell regulatory genes and metastasis-related genes in U-87 MG and LN-229 cells. The results demonstrated that the protein levels of CDK4, CDK6, CyclinD1, N-cadherin, and c-Myc were significantly reduced in RANBP10-downregulation GBM cells, whereas p21 and E-cadherin were increased in RANBP10-knockdown groups (Fig. 4A). In addition, the mRNA levels of all these genes were significantly altered in the RANBP10-downregulation GBM cells, except c-Myc (Fig. 4B). Interestingly, the GESA analysis using the CGGA database indicated that RANBP10 high expression was positively associated with c-Myc target genes in glioma (Supplementary Fig. S7). Thus, we speculated that RANBP10 might regulate c-Myc expression post-transcriptionally. Indeed, RANBP10 mediated c-Myc downregulation could be rescued by the proteasome inhibitor MG132 (Fig. 4C and Supplementary Fig. S8). Moreover, the turnover rate of c-Myc protein was examined and the results indicated that RANBP10 overexpression could reduce the turnover rate of c-Myc (Fig. 4D, E). Then, we performed the ubiquitination assay and found that RANBP10 overexpression significantly reduced c-Myc ubiquitination in U-87 MG and LN-229 cells (Fig. 4F). Collectively, these data suggested that RANBP10 elevated c-Myc protein stability by reduction of c-Myc ubiquitination.

### RANBP10 suppressed the transcription of FBXW7

**Fig. 5:**
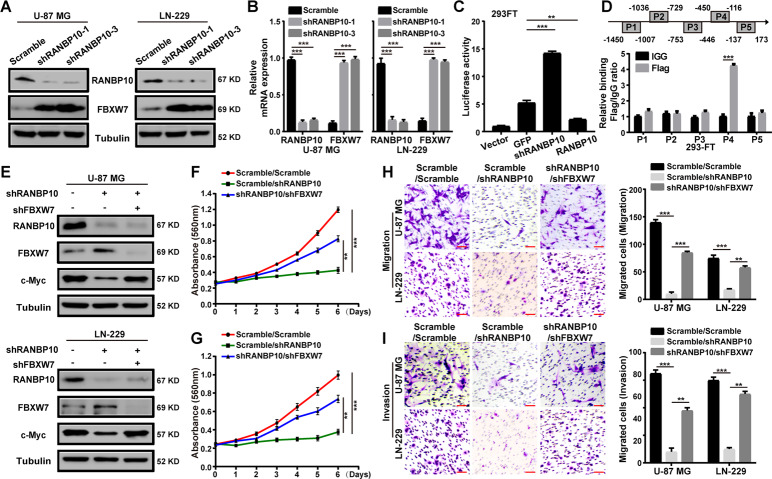
RANBP10 suppressed the transcription of FBXW7. **A** Western blot analysis was used to detect the protein expression of FBXW7 in RANBP10 downregulation and control cells. **B** RT-PCR analysis was used to detect the mRNA expression of FBXW7 in RANBP10 downregulation and control cells. **C** Dual-luciferase reporter assays were used to detect the promoter activity of FBXW7. **D** ChIP assay was performed by using Flag antibodies. IgG was used as the negative control. **E** Western blot analysis was performed to examine the protein expression of FBXW7 and c-Myc in the indicated cells. **F**, **G** MTT assays were used to assess the proliferative effects of FBXW7 downregulation in RANBP10-knockdown and control cells. **H**, **I** Transwell assays were used to assess the metastatic effects of FBXW7 downregulation in RANBP10-knockdown and control cells. Scale bar = 50 μm. The data were expressed as mean ± SD. Student’s *t* test was performed to analyzed significance. *P < 0.05, **P < 0.01, ***P < 0.001.

RANBP10 has been reported as a transcription regulator in human cancer [13]. FBXW7 acts as the main E3 ubiquitin ligase for the ubiquitination and degradation of c-Myc. Thus, we speculated that RANBP10 might regulate c-Myc protein stability by targeting FBXW7. We analyzed the data from the CGGA database and revealed the negative correlation of RANBP10 and FBXW7 in glioma (Supplementary Fig. S9A). Then, western blot and RT-PCR analyses were performed and demonstrated that FBXW7 expression was increased at the protein and mRNA levels in RANBP10-knockdown groups (Fig. 5A, B). To determine whether RANBP10 regulate FBXW7 expression at the transcription level, the dual-luciferase reported assay was performed and showed that the promoter activity of FBXW7 was increased in the RANBP10-knockdown group and was decreased in the RANBP10-overexpression group, suggesting that RANBP10 inhibited the FBXW7 promoter activity (Fig. 5C). To further determine whether RANBP10 binds the promoter of FBXW7, we performed the ChIP assay and found that RANBP10 bound the region P4 (−450 to −116 bp) of the FBXW7 promoter (Fig. 5D). To determine whether RANBP10 reduces c-Myc ubiquitination by negatively regulates the FBXW7 expression, the ubiquitination assay was performed and revealed that RANBP10 overexpression could decrease the FBXW7-mediated c-Myc ubiquitination (Supplementary Fig. S9B). To further confirm that RANBP10 modulated c-Myc protein stability by targeting FBXW7, we treated the RANBP10-downregulation GBM cells with the highly effective shFBXW7 sequence (Supplementary Fig. S9C) [23]. Western blot analysis was performed and confirmed that the expression of c-Myc was significantly increased after FBXW7-downregulation treatment (Fig. 5E). Consistently, cell proliferation, migration, invasion of RANBP10-knockdown cells was dramatically increased after FBXW7-knockdown treatment (Fig. 5F–I). Collectively, these data indicated that FBXW7 was an important downstream effector of RANBP10 in GBM.

### RANBP10 promoted tumor growth of GBM cells

**Fig. 6:**
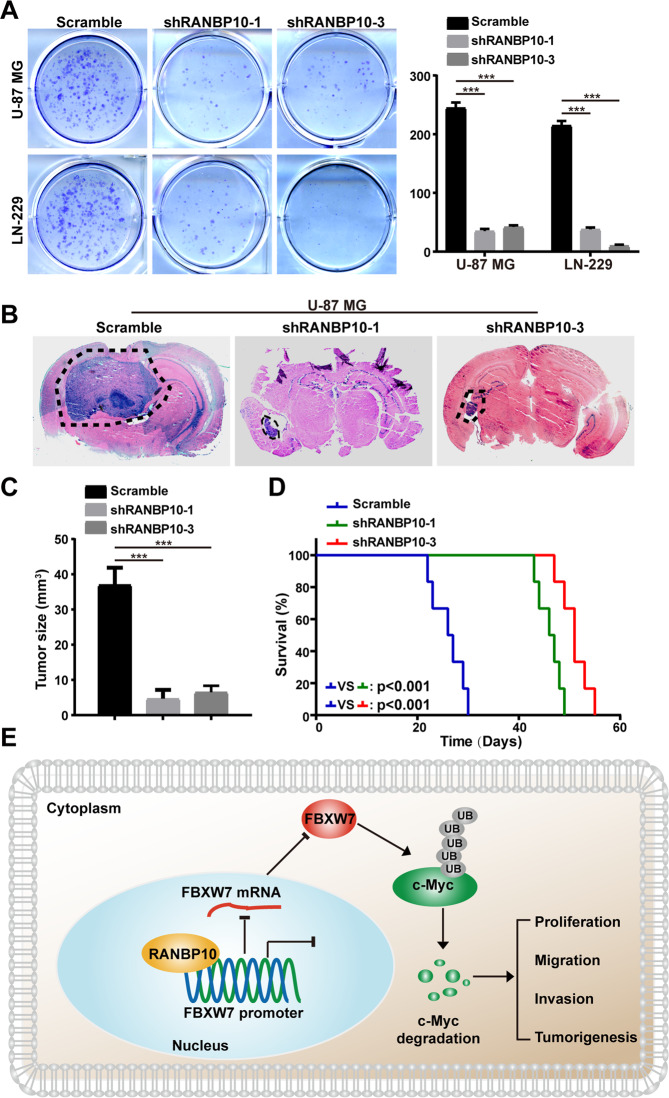
RANBP10 promoted tumor growth of GBM cells. **A** Colony-formation assays were performed to examine the in vitro tumor formation capabilities of RANBP10-knockdown and control cells. **B**, **C** Orthotopic implantation experiment was performed to assess the in vivo tumor formation capabilities of RANBP10-knockdown and control cells. **D** Overall survival of RANBP10-knockdown and control mice and *P* value is indicated. **E** Model of the impact of the RANBP10 on regulating cell proliferation, migration, invasion, and tumorigenesis of GBM cells. The data were expressed as mean ± SD. Student’s *t* test was performed to analyzed significance. *P < 0.05, **P < 0.01, ***P < 0.001.

To explore the functional role of RANBP10 in tumor growth of GBM cells, in vitro colony-formation assay was performed and indicated that silencing of RANBP10 clearly inhibited the colony formation of U-87 MG and LN-229 cells (Fig. 6A). In addition, orthotopic implantation assay was performed and demonstrated that RANBP10 downregulation dramatically retarded the tumor formation capabilities of U-87 MG cells (Fig. 6B, C). Consistently, the overall survival of RANBP10-knockdown groups was clearly improved compared with the control groups (Fig. 6D). Taken together, these results indicated that RANBP10 promoted colony formation and tumor growth of GBM cells by targeting the FBXW7/c-Myc signaling pathway (Fig. 6E).

## Discussion

GBM is a highly aggressive kind of brain neoplasm, with only a median survival of 12-15 months and a 5-year survival rate of <5% [25]. Perhaps the grim prognosis is mainly due to the poor understanding of the molecular mechanism of GBM development and progression. Thus, better investigating GBM progression and further identifying molecular biomarkers for prognosis are crucially needed to improve GBM patient overall survival. Our results revealed that RANBP10 was overexpressed in GBM, and patients with high RANBP10 expression showed poor prognosis, suggesting that RANBP10 may act as a tumor promoter in GBM. To determine whether RANBP10 promoted GBM progression, we knocked down RANBP10 using the shRNA sequences and found that downregulation of RANBP10 significantly inhibited cell proliferation, migration, invasion, and tumor growth of GBM cells.

RANBP10 was originally identified as a paralog of RANBP9 in the human genome. RANBP9, also known as RANBPM, has been found to directly or indirectly interact with some proteins involved in a wide variety of biological progression [26, 27]. In addition, RANBP9 had an inhibitory or primitive regulatory effect on gene transcription, such as AβPP intracellular domain (AICD) and AR [28, 29]. Mounting evidence have demonstrated that RANBP9 acted as an important mediator of DNA damage response (DDR), cell proliferation, migration, invasion, and apoptosis in human cancers [30, 31]. Unlike RANBP9, the biological functions and roles of RANBP10 in human tumors are poorly understood. Extensive protein similarities suggest that RANBP10 might have partially overlapping functions with RANBP9. For instance, RANBP10 has been reported to be a Ran-binding and β-tubulin-binding protein and to facilitate nuclear transport and microtubule organization in megakaryocytes [7, 8, 32]. Increasing evidence has indicated that RANBP10 was involved in multiple functions via its interaction with various proteins [32]. In addition, RANBP10 also reported to function as a transcription mediator for the androgen receptor (AR) and several viral genes [12, 13]. In AR-positive prostate cancer cells, RANBP10 formed a protein complex with itself or RANBP9 and then elevated the transcription activity of AR. Furthermore, Yuka et al. reported that RANBP10 and herpes simplex virus 1 (HSV-1) regulatory protein ICP0 redundantly and synergistically regulated viral gene expression and replication by modulating chromatin remodeling [12]. Thus, RANBP10 might form a protein complex with other proteins and then transcriptionally regulate gene expression.

In our study, we performed the GESA analysis using the CGGA database and found that RANBP10 high expression was positively associated with c-Myc target genes in glioma. Furthermore, we found that RANBP10 was responsible for the protein stability of c-Myc in GBM cells. Through the dual-luciferase reported assay, we showed that the transcription activity of FBXW7, the main E3 ubiquitin ligase of c-Myc, was clearly inhibited by RANBP10. In addition, we found that RANBP10 bound the region P4 of FBXW7 promoter through the ChIP analysis. The ubiquitination assay was performed and revealed that RANBP10 overexpression could decrease the FBXW7-mediated c-Myc ubiquitination.

In conclusion, our data indicated that RANBP10 promoted cell proliferation, migration, invasion, and tumor growth of GBM cells. In addition, we found that RANBP10 played a critical mediator of c-Myc protein stability by inhibition of FBXW7-transcriptional activity. These findings offer new insights into the biological function of RANBP10 and suggest that RANBP10 serves as a promising target in GBM therapy.

## Supplementary information

Supplementary meterial files

Figure-S1

Figure-S2

Figure-S3

Figure-S4

Figure-S5

Figure-S6

Figure-S7

Figure-S8

Figure-S9

## Data Availability

All of the data and material in this paper are available when requested.
