# Supplementary material for: RANBP10 promotes glioblastoma progression by regulating the FBXW7/c-Myc pathway
Source: Cell Death Dis. 2021 Oct 20;12(11):967. doi: 10.1038/s41419-021-04207-4 (PMC8528885; doi:10.1038/s41419-021-04207-4)
Supplement: Supplementary file 1 — Supplementary meterial files [file 41419_2021_4207_MOESM1_ESM.docx]

**Figure legends**

**Supplementary Fig. 1 (A)** Level of RANBP10 in malignant tumors were analyzed using data from Oncomine database. (B) Expression of RANBP10 in malignant tumors were analyzed using data from the Human Protein Altas database.

**Supplementary Fig. 2** Box plot of RANBP10 expression levels in Murat Brain (Oncomine) database with the log-rank test P-values indicated.

**Supplementary Fig. 3** Flow cytometric analysis of cell apoptosis in RANBP10-downregulation and control cells.

**Supplementary Fig. 4** Flow cytometric analysis of cell cycle distribution in RANBP10-downregulation and control cells.

**Supplementary Fig. 5** Cell invasion of RANBP10-knockdown and control cells were examined by transwell assay. Scale bar=50 μm. The data were expressed as mean ± SD. Student’s t- test was performed to analyzed significance. *P<0.05, **P<0.01, ***P<0.001.

**Supplementary Fig. 6** Transwell assays were used to detect the effect of RANBP10 overexpression on the invasion of RANBP10-knockdown and control cells. Scale bar=50 μm. The data were expressed as mean ± SD. Student’s t- test was performed to analyzed significance. *P<0.05, **P<0.01, ***P<0.001.

**Supplementary Fig. 7** GSEA enrichment analysis of c-Myc target gene signatures in RANBP10 high expression versus RANBP10 low expression CGGA gliomas, Normalized enrichment score (NES), P-values, and false discovery rate (FDR) were indicated.

**Supplementary Fig. 8** RANBP10-knockdown cells were treated with or without MG132 for 8 h before harvesting. Western blot assays were performed to detect the protein expression of CDK4, N-cadherin, CDK6 and CyclinD1.

**Supplementary Fig. 9** (A) Analysis of expression data from CGGA database revealed that RANBP10 level was negatively correlated FBXW7 level in glioma. (B) The indicated plasmids were transfected into GBM cells, and MG132 was added to the cells before harvested. The ubiquitinated c-Myc proteins were pulled down with anti-HA antibody and immunoblotted with ant-c-Myc antibody. (C) The expression of FBXW7 protein was detected by western blot analysis.
