# Supplementary figures and images for: RANBP10 promotes glioblastoma progression by regulating the FBXW7/c-Myc pathway
Source: Cell Death Dis. 2021 Oct 20;12(11):967. doi: 10.1038/s41419-021-04207-4 (PMC8528885; doi:10.1038/s41419-021-04207-4)

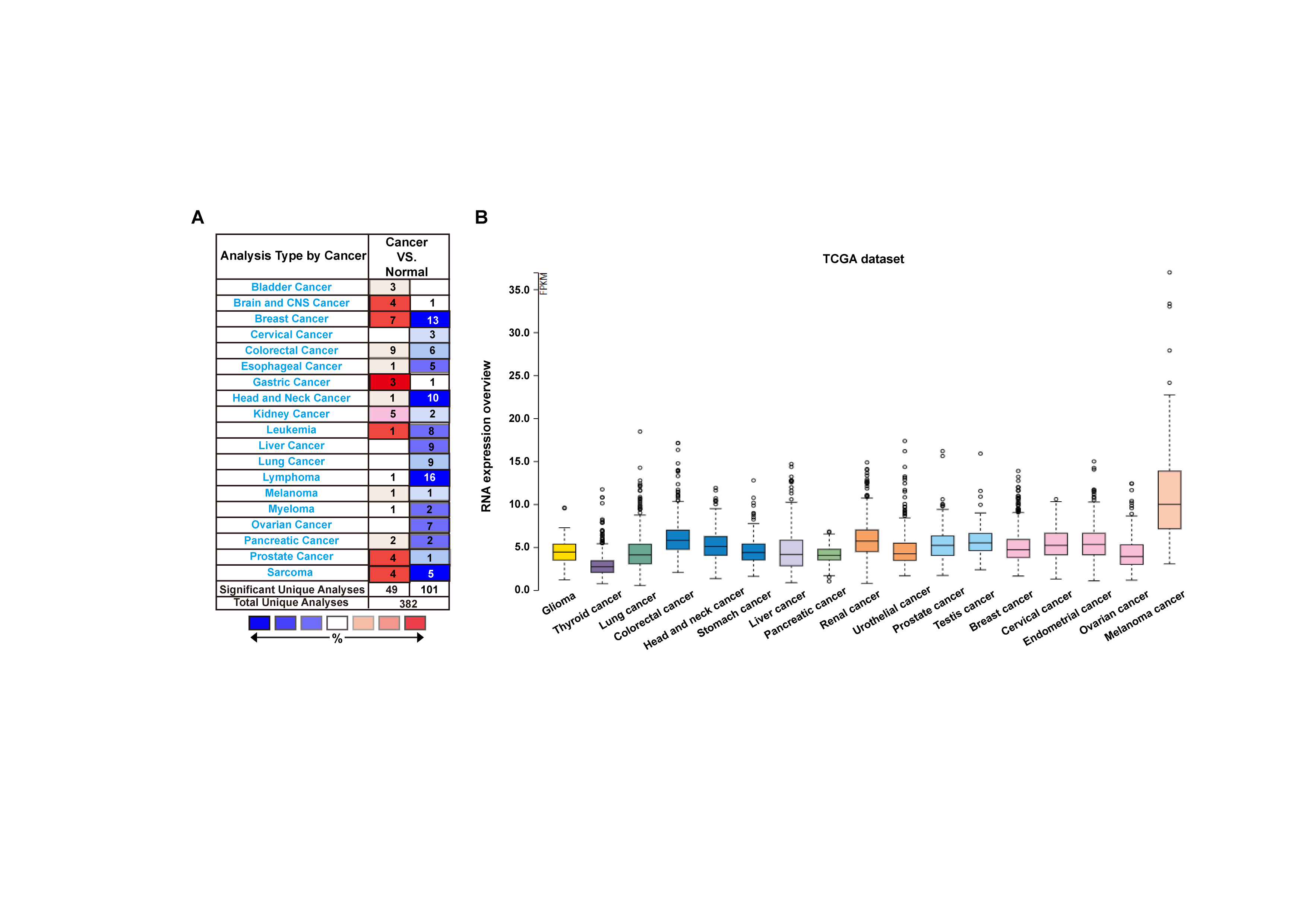

Supplement: Supplementary file 2 — Figure-S1 [file 41419_2021_4207_MOESM2_ESM.tif]

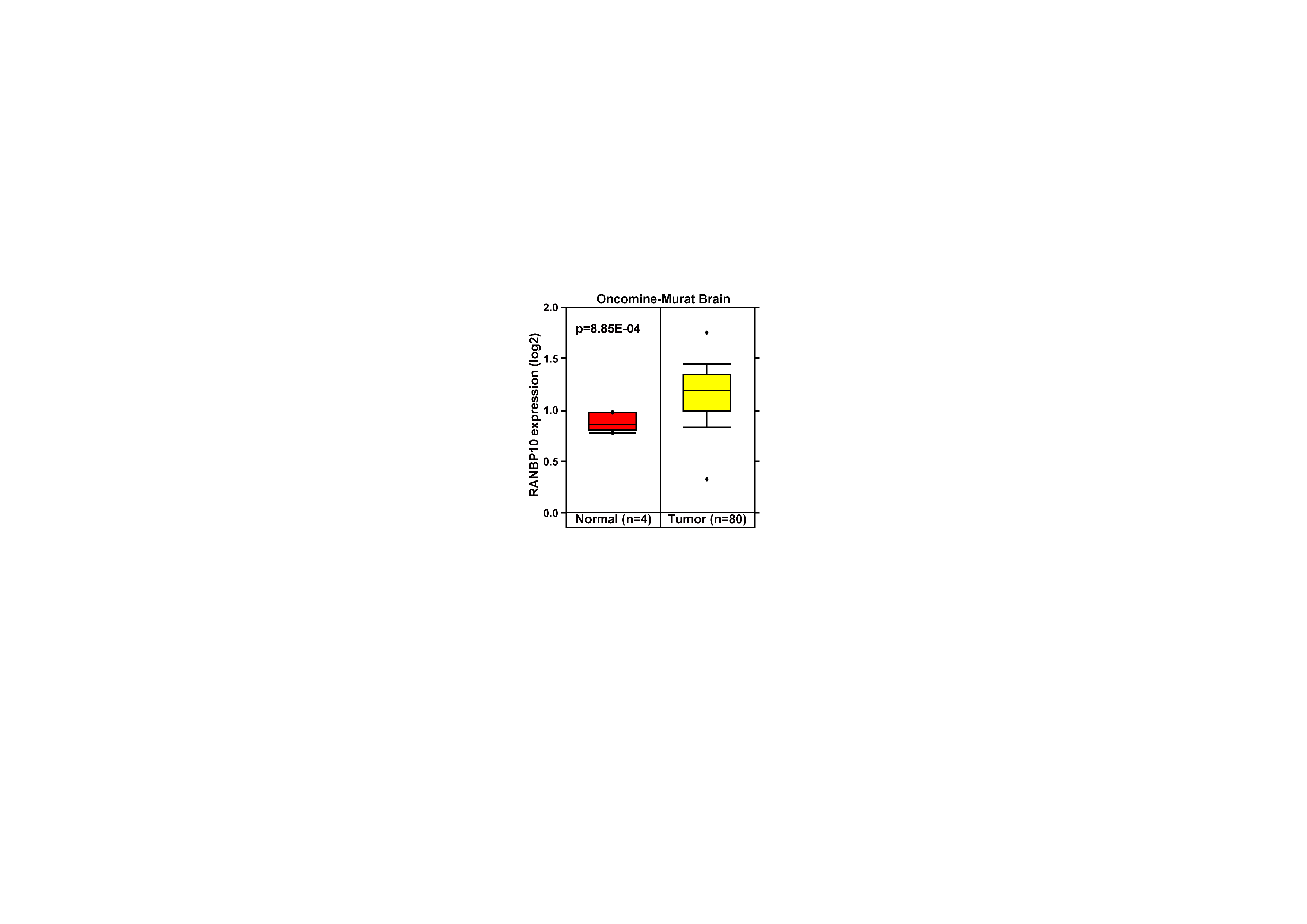

Supplement: Supplementary file 3 — Figure-S2 [file 41419_2021_4207_MOESM3_ESM.tif]

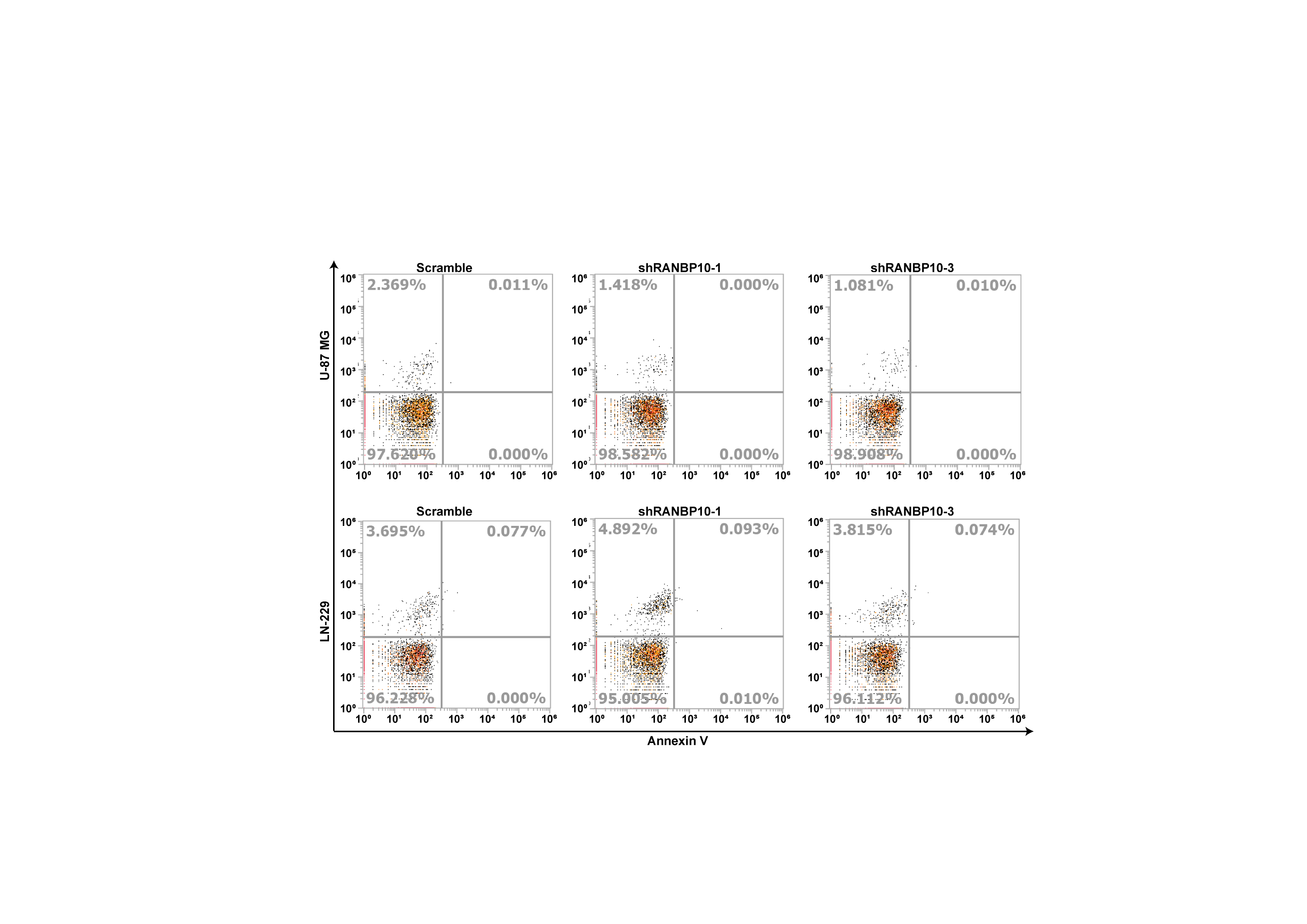

Supplement: Supplementary file 4 — Figure-S3 [file 41419_2021_4207_MOESM4_ESM.tif]

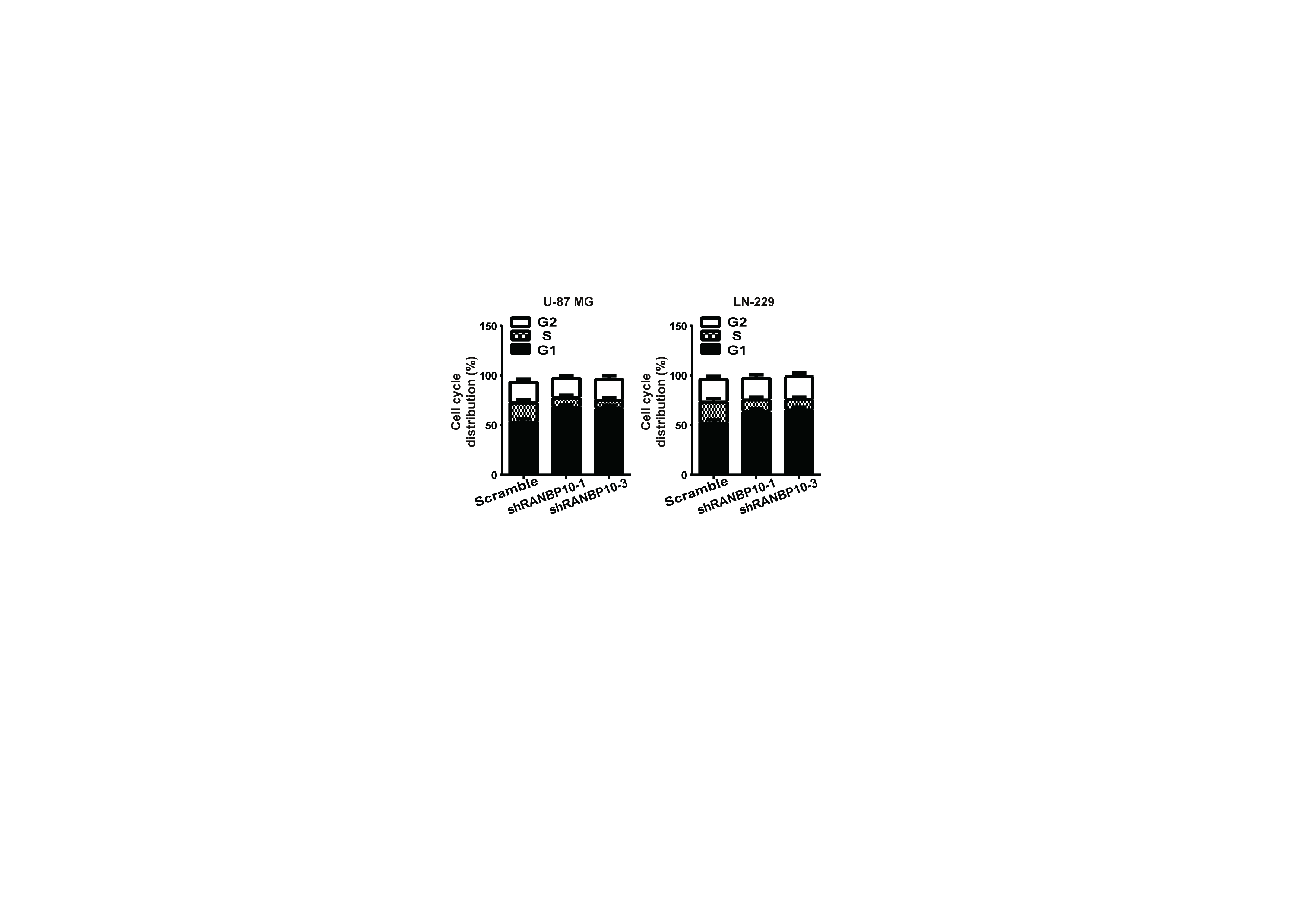

Supplement: Supplementary file 5 — Figure-S4 [file 41419_2021_4207_MOESM5_ESM.tif]

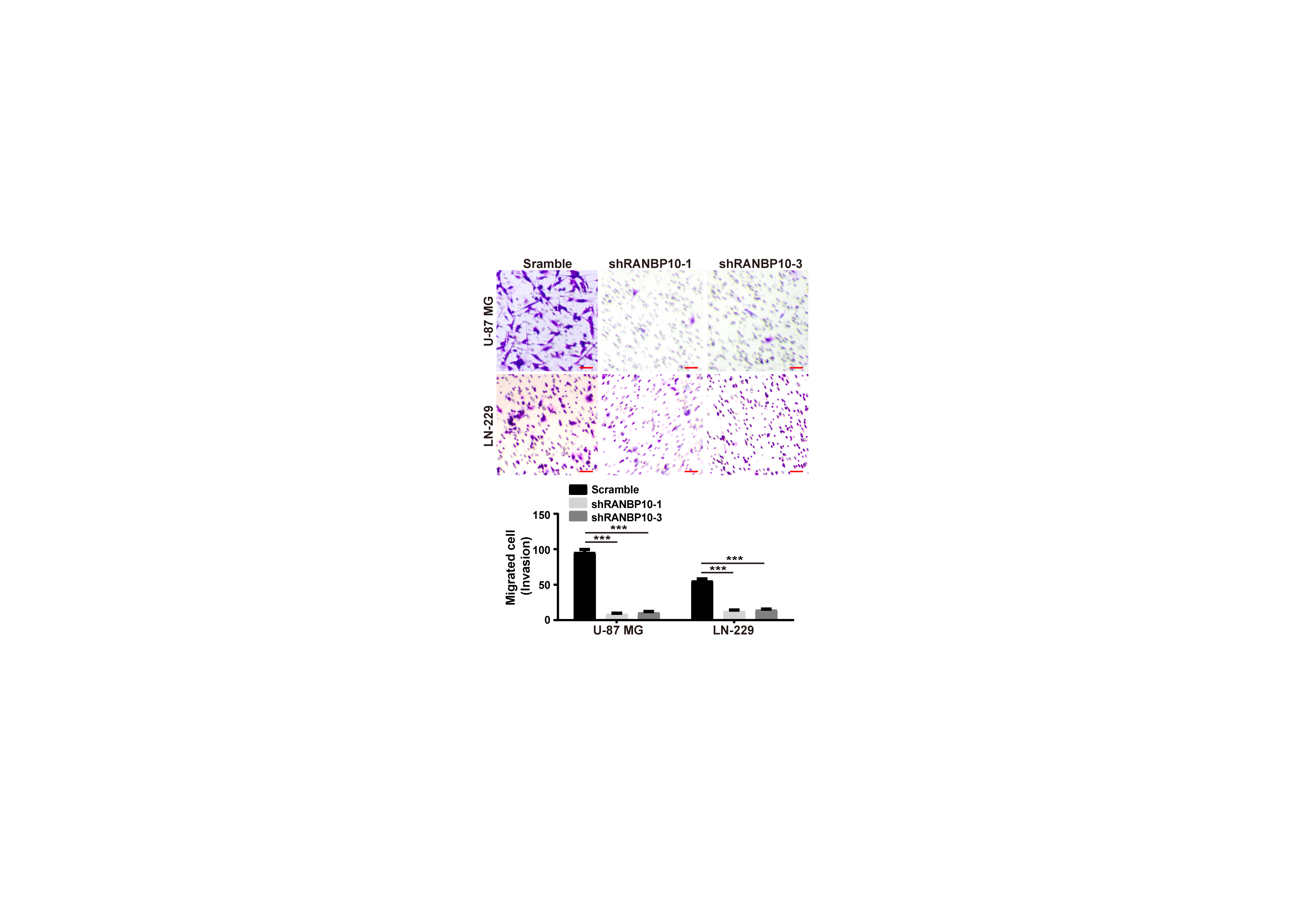

Supplement: Supplementary file 6 — Figure-S5 [file 41419_2021_4207_MOESM6_ESM.tif]

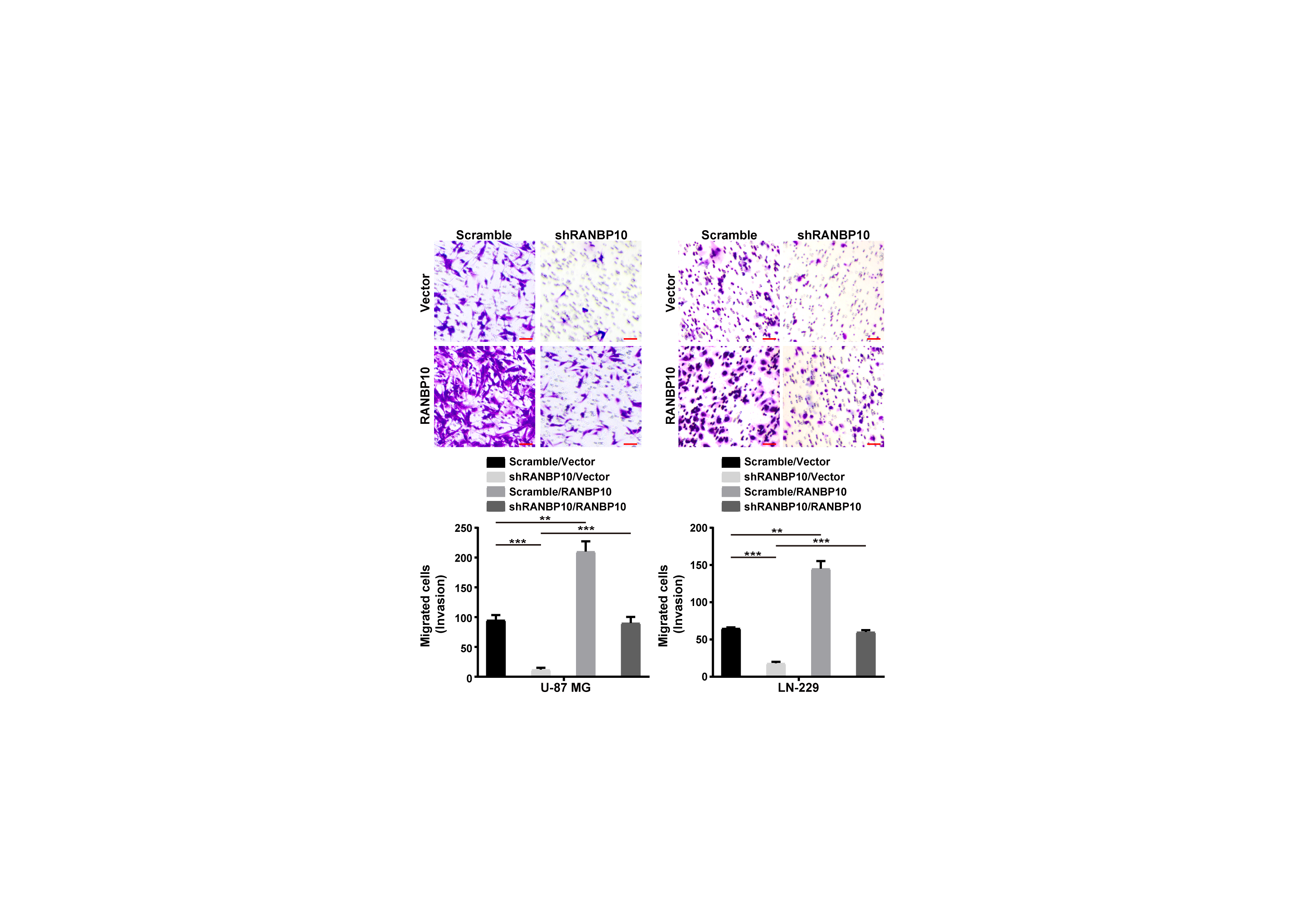

Supplement: Supplementary file 7 — Figure-S6 [file 41419_2021_4207_MOESM7_ESM.tif]

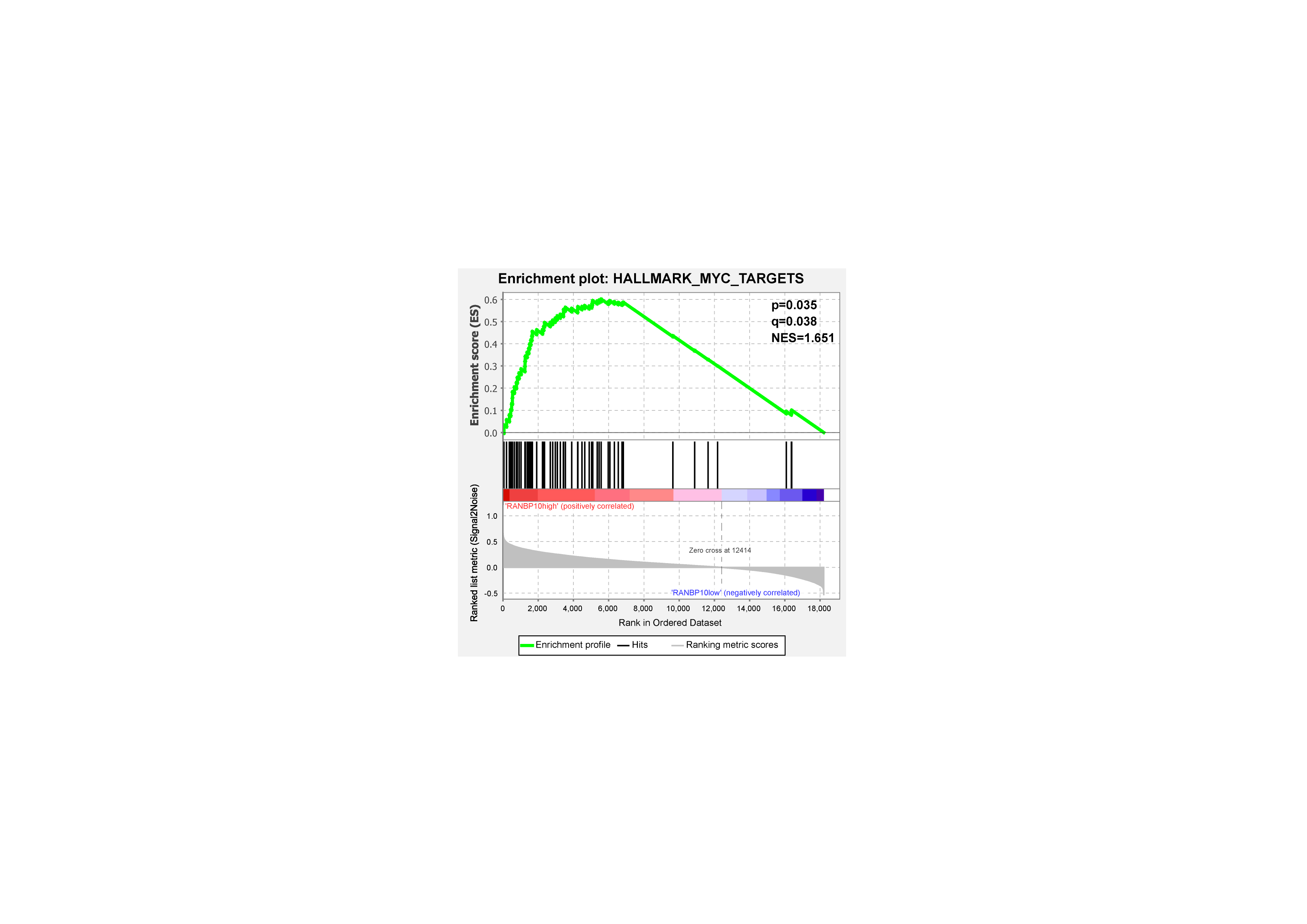

Supplement: Supplementary file 8 — Figure-S7 [file 41419_2021_4207_MOESM8_ESM.tif]

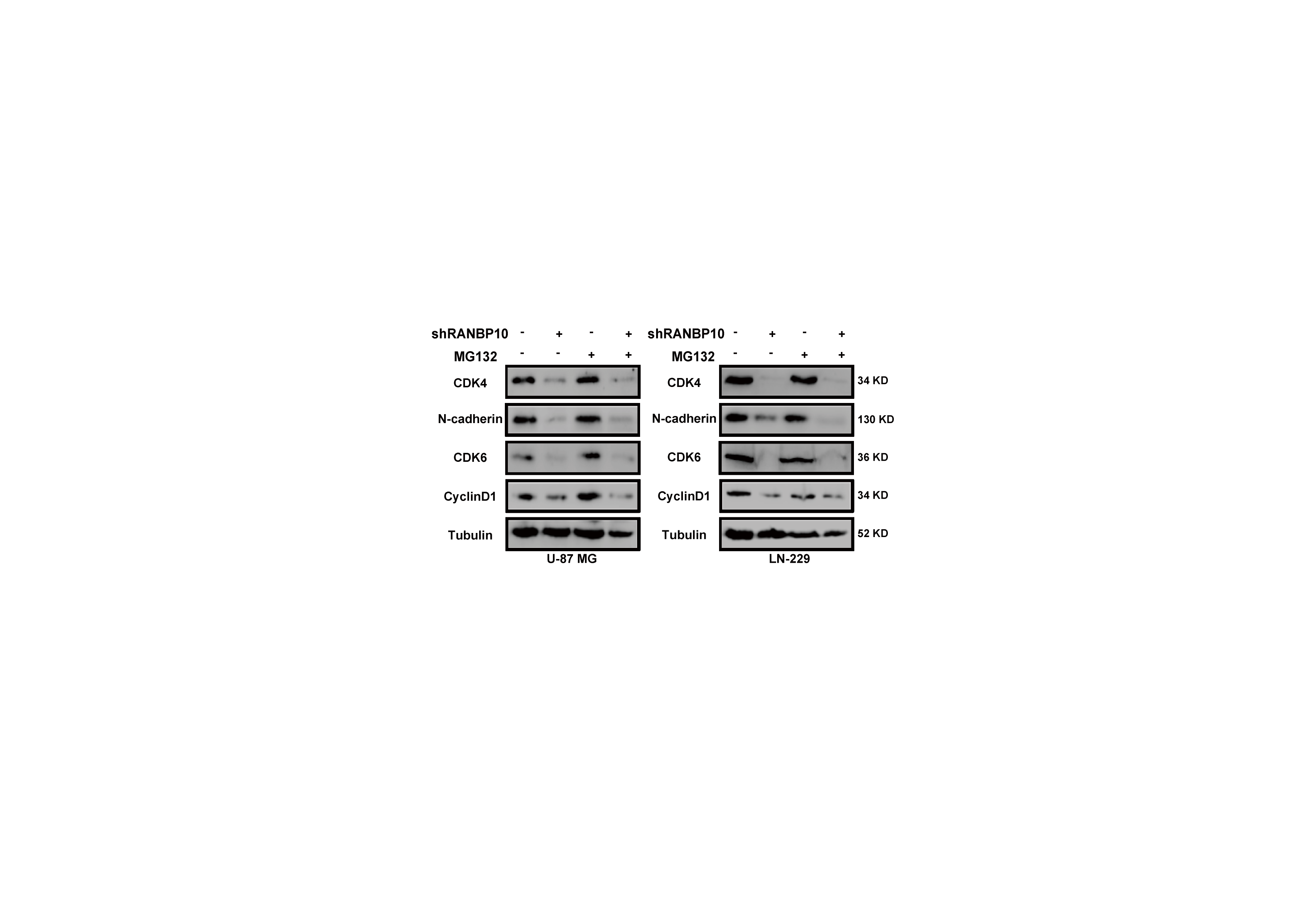

Supplement: Supplementary file 9 — Figure-S8 [file 41419_2021_4207_MOESM9_ESM.tif]

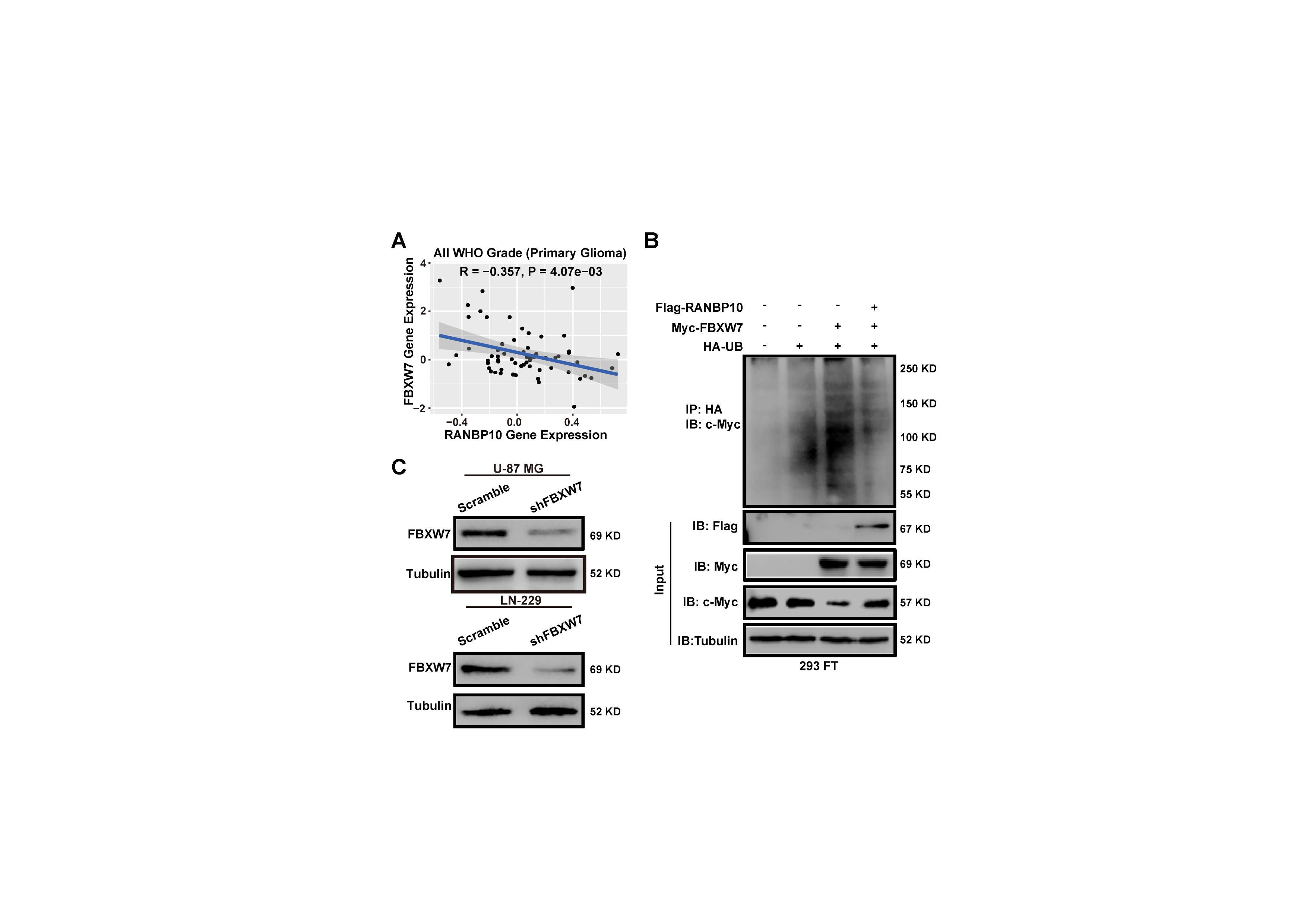

Supplement: Supplementary file 10 — Figure-S9 [file 41419_2021_4207_MOESM10_ESM.tif]
